# Medical Image Data and Datasets in the Era of Machine Learning—Whitepaper from the 2016 C-MIMI Meeting Dataset Session

**DOI:** 10.1007/s10278-017-9976-3

**Published:** 2017-05-17

**Authors:** Marc D. Kohli, Ronald M. Summers, J. Raymond Geis

**Affiliations:** 1 Radiology and Biomedical Imaging, 505 Parnassus Ave, Moffit-391, San Francisco, CA 94117 USA; 2 Imaging Biomarkers and Computer-Aided Diagnosis Laboratory, Radiology and Imaging Sciences, Clinical Center, National Institutes of Health, Bethesda, MD 20892-1182 USA; 3 University of Colorado School of Medicine, 3401 Shore Rd, Fort Collins, CO 80524 USA

**Keywords:** Machine learning, Medical imaging, Imaging informatics, Medical data, Radiology, Medical image datasets

## Abstract

At the first annual Conference on Machine Intelligence in Medical Imaging (C-MIMI), held in September 2016, a conference session on medical image data and datasets for machine learning identified multiple issues. The common theme from attendees was that everyone participating in medical image evaluation with machine learning is data starved. There is an urgent need to find better ways to collect, annotate, and reuse medical imaging data. Unique domain issues with medical image datasets require further study, development, and dissemination of best practices and standards, and a coordinated effort among medical imaging domain experts, medical imaging informaticists, government and industry data scientists, and interested commercial, academic, and government entities. High-level attributes of reusable medical image datasets suitable to train, test, validate, verify, and regulate ML products should be better described. NIH and other government agencies should promote and, where applicable, enforce, access to medical image datasets. We should improve communication among medical imaging domain experts, medical imaging informaticists, academic clinical and basic science researchers, government and industry data scientists, and interested commercial entities.

## Introduction

The first annual Conference on Machine Intelligence in Medical Imaging (C-MIMI) was held on September 12–13, 2016, in Alexandria, Virginia, under the auspices of the Society for Imaging Informatics in Medicine (SIIM). This paper summarizes a conference session which discussed medical image data and datasets for machine learning. It also reviews unique domain issues with medical image datasets.

The amount and quality of training data are dominant influencers on a machine learning (ML) model’s performance. The common theme from all attendees was that everyone participating in medical image evaluation with machine learning is data starved. This is a particularly pressing problem in the new era of deep learning [1]. There is an urgent need to find ways to collect and reuse medical imaging data. Beyond this universal primary sentiment, the session identified a diverse set of challenges, described here.

## The Ideal Dataset for Medical Imaging Machine Learning

The ideal medical image dataset for an ML application has adequate data volume, annotation, truth, and reusability. At base, each medical imaging data object contains data elements, metadata, and an identifier. This combination represents an “imaging examination.” A collection of data objects or dataset must have enough imaging examinations to answer the question being asked. To maximize algorithm development, both the dataset itself and each imaging examination must be described and labeled accurately. Ground truth, the classification label(s) of each imaging examination, should be as accurate and reproducible as possible. Furthermore, an ideal dataset is Findable, Accessible, Interoperable, and Reusable (FAIR) [2]. In the coming sections, we will describe features of machine learning datasets.

## Use Cases

Use case is a computer science term that defines actions or steps performed by an actor and a system to achieve a particular goal [3]. When it comes to medical imaging ML, we tend to think of use cases as clinical questions: Can image texture be used to predict MGMT methylation status of brain tumors [4], or can we distinguish frontal from lateral chest radiographs? [5] Other use cases could be administrative, prioritize work within a queue, or otherwise facilitate machine and/or human workflow.

An ideal use case defines a project which is specific, measurable, and achievable, and has well-defined users and value. Use cases also help to illustrate standards, or the need for standards. Creating a good ML proposal is similar to creating a good research question—it should be carefully designed, and measurable.

Use cases can be made for medical imaging datasets themselves: appropriately annotated datasets that can be used to train, test, validate, and verify machine learning algorithms. The value to industry, academia, and the public is that machine learning algorithms will improve healthcare outcomes and reduce healthcare costs. Customers of these datasets will be industry, academic, and governmental researchers and regulatory bodies. Each customer will have different aims including building machine learning products, gaining new insights from medical images beyond human perception, and understanding how to verify and regulate machine learning products that consume and process medical image data.

Use cases for medical ML datasets vary considerably. Academicians may be satisfied with small, tightly focused datasets to efficiently answer focused questions that result in publications and further funding. In contrast, industry desires data of sufficient volume, variety, and quality to make ML models that work in disparate online production environments. Commercial medical image ML products will need independent verification and potential FDA approval. Verification datasets should include data elements that stress or even break an ML product, to understand its limits, biases, and even potential ethical issues. The larger data science and business analytics communities may federate and mine these datasets for new insights. As the value of these datasets becomes better appreciated, data repositories and data publishing entities, whether public, commercial, or academic, will develop business cases around these datasets. Machines will define their own use cases; over time, this scenario may be the prime originator of new use cases.

Machine learning competitions, such as those hosted on http://kaggle.com, may define distinctive dataset use cases depending on competition goals. Datasets may be close to their raw form, or in other cases transformed into an anonymized feature matrix easily consumed by standard supervised machine learning algorithms. Competitions that use medical image datasets typically transform data: limit to a relevant subset of rows, alter or remove columns, choose an error metric, split the data into train/validation/test sets, and try to identify and mitigate potential sources of data leakage. In the next section, we will shift to describing metadata important to machine learning datasets.

## Dataset Specifications—Metadata

Metadata for medical imaging ML include data generated by an imaging modality, prescribed exam codes and description data based on an order, and annotations indicating the content and/or anatomy of a particular image. Table 1 shows examples of commonly available metadata elements. As shown in Table 1, there are a variety of original sources of metadata and associated storage mechanisms. There are a variety of challenges to making metadata easily understood by a computer or algorithm. These challenges arise at both the institutional level and when sharing data between institutions.

**Table 1:** Examples of commonly available metadata

Element	Source	Example	Storage location
PatientsName	EHR/ADT	MARY^JONES^B	DICOM header
PatientID	EHR/ADT	1232391-3	DICOM header
StudyDescription	RIS	CT BRAIN W/O	DICOM header
Rows	Imaging modality	512	DICOM header
Columns	Imaging modality	512	DICOM header
BitsStored	Imaging modality	12	DICOM header
Key Images	Radiologist		DICOM Key Object
Measurement	Radiologist		Various (AIM, DICOM PS, DICOM SR)

*ADT* admission, discharge, and transfer, *EHR* electronic health record, *RIS* radiology information system

Consider the procedure description for an MR study performed to evaluate rectal cancer staging. Today, some centers would consider this an MRI Pelvis Without Contrast, and other centers would have a more specific description such as MRI Rectal Cancer Staging. It is relatively easy for a human reading a radiology report to identify these discrepancies. Furthermore, it is likely that the centers that have more specific study descriptions started with something more generic, so the task of retrieving all studies related to rectal cancer screening from an archive requires institutional knowledge, and takes longer (and costs more) than one might expect.

An additional wrinkle with exchanging data across institutions is study description heterogeneity between institutions. This can be addressed by asking all institutions to utilize institutional knowledge to map local study descriptions to the RadLex playbook [6]. This type of mapping is reasonable for focused use cases, but becomes unwieldy when trying to build larger federated datasets without automated tools [7].

## Dataset Specifications—Metadata—DICOM Header

Digital Imaging and Communications in Medicine (DICOM) is the standard file format definition and communication profile for radiological and many other medical images. The DICOM file format contains required and optional metadata fields which describe the patient, exam details, and, in many cases, individual image details. The individual image details described in DICOM metadata typically relate to technical aspects of the image (e.g., rows, columns, modality, manufacturer) rather than an inclusion of a particular organ, or diagnosis. The major benefit of DICOM is that it provides a standard for medical image storage and a set of network operations for transmission and retrieval. In practice, however, data fields may be filled incorrectly or left blank, some with interoperable and others with proprietary data [8]. While DICOM issues usually do not interfere with clinical image viewing, they may cause complex problems when federating datasets. In some instances, de-identifying datasets may result in the removal of metadata that is required for advanced processing. In competitions, occasionally DICOM metadata quirks improve algorithm performance, but are generalizably applicable outside the competition.

## Dataset Specifications—Metadata—Private Tags

DICOM allows private data elements, and these are commonly used by individual OEMs and individual healthcare institutions to enable distinctive workflow. Occasionally, these include personal health information (PHI), pathology-related identifiers, or specific research or clinical protocols.

The DICOM standard is rich with specifications that are underutilized by vendors. One such specification is structured reporting (DICOM-SR) [9–11]; hopefully, this will come into greater use in the future for storing a particular type of metadata—annotations.

## Dataset Specifications—Metadata—Annotations

**Fig. 1:**
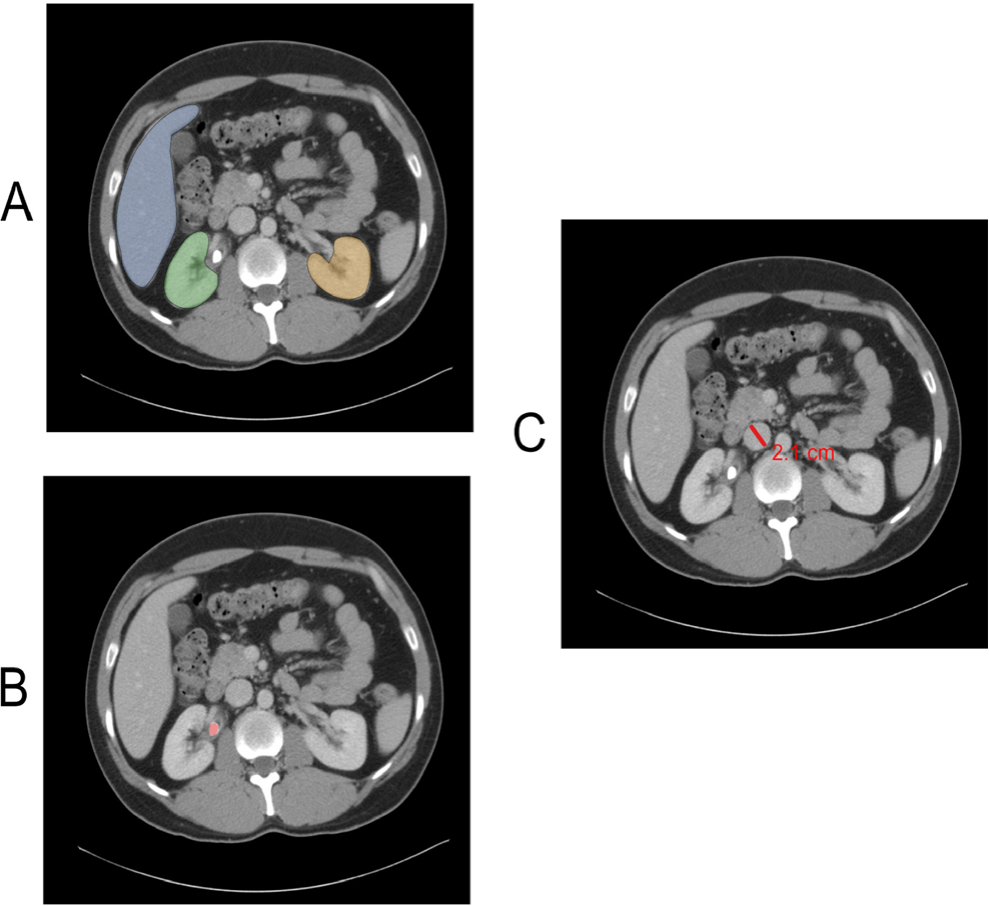
Three different types of image annotations: anatomic region of interest segmentations (**a**), pathology region of interest segmentation such as this urinary calculus (**b**), and measurements (**c**)

For this paper, we consider annotations as a special class of metadata that pertain to the diagnoses, or anatomical or pathological regions contained within a particular image (Fig. 1). Challenges with annotations fall into main two categories: interoperability and ground truth.

Computer-based image recognition and analysis require high-quality, annotated datasets that cover the entities described in the use case, including normal cases and those with pathology ranging from very subtle findings to very severe. Well-annotated datasets with representative label distribution are crucial to training accurate, generalizable models. Additionally, due to the large volume of image data required for many ML projects, new annotation tools focused on efficiency and rapid annotation are needed. Keen researchers will focus on producing datasets in a manner that allows their robust use in algorithm development, validation, and certification.

Unfortunately, and not without effort by several parties, there is no universally applied syntax, format, or semantic for radiologic image annotation and markup. Commercial vendors use proprietary annotations and markups, few of which are interoperable. The Annotation and Image Markup (AIM) standard has been successful as a research tool, but has not been widely implemented clinically [12]. DICOM presentation state objects are a competing standard for storing and transmitting annotations, which provides less functionality than AIM. Currently, this means that measurements or regions of interest made in one vendor’s workstation will not appear when the images are moved and viewed on a different vendor’s viewing platform, and labels may have different meanings or definitions without use of some unified or standardized vocabulary. Annotations overlaid on images may include PHI, clinical or pathology information, arrows, question marks, or other potentially important but not easily discoverable or understandable data.

Robust solutions to segment anatomic structures are now available for many organs, but the process is not standardized. Segmentation annotations are rarely transferable among vendors. Standards to document how a segmentation was performed do not exist, or are proprietary.

Universally accepted methods to annotate ground truth of medical images have not been developed. Where different definitions of ground truth are used, the advantages and disadvantages of labeling methods are yet to be evaluated. The process of ground truth annotation currently includes radiologists’ reports, pathology reports, surgical reports, crowdsourced annotation, and clinical follow-up. Depending on the clinical question, clinical follow-up may be from weeks to years.

In practice, medicine is more ambiguous than commonly presented. Many features found in any single image exist on a continuum. Thus, throughout medicine, clusters of findings are arbitrarily grouped into categories, such as “normal,” “not quite normal,” “suspicious for being abnormal,” or “definitely abnormal.” Cascades of these types of grading systems interact with each other. For example, mammogram findings are grouped into seven categories, from 0 to 6, depending on the degree of suspicion. Category 4 is suspicious findings that may require biopsy. In turn, pathology description of the biopsy is similarly categorized along a spectrum of multiple categories.

Radiologists’ reports are not definitive expressions of ground truth [13–16]. A retrospective 20-year literature review in 2001 suggested that the level of significant radiology error ranged between 2 and 20% [17]. This is not limited to radiology; a Mayo clinic study comparing clinical diagnoses with postmortem autopsies reported that a major diagnosis was missed clinically in 26% of patients [18].

Even using radiology reports to annotate “normal,” or “no disease,” is problematic. For example, one study showed that 90% of lung cancers detected on radiographs were visible in retrospect on previous examinations [19]. This startling result did not mean radiologists were terrible. Rather, in some cases, lesions retrospectively noted on original exams were small, indistinct, or otherwise had features of normal structures or incidental findings. Even larger lesions are missed, however; a review of non-small cell lung cancer diagnosed on radiographs showed that 19% of lesions were missed, and the median diameter of missed nodules was 16 mm [20].

## Dataset Specifications—Pixel Data

Historically, images used in object recognition ML have relatively small matrix size, to decrease computation time. In contrast, medical images often have very high dimensionality. In clinical practice, radiology image matrices may vary from 64 × 64 for some nuclear medicine exams, to over 4000 × 5000 for some mammogram images. CT and MRI scans, which typically are 512 × 512 and 256 × 256, respectively, for each image, may have well over 1000 images per exam. The number of extracted features may be very large. In order to train a network fully, each algorithm parameter requires at least several pixels to compute features.

Radiographs and fluoroscopic pixels represent 2D information, while CT, MR, PET, and ultrasound pixels represent 3D voxels. Some exams acquire time-dependent, 4D, data. Nuclear medicine has multi-dimensional, multi-frame images. Among many variables, voxel characteristics may vary based on patient characteristics such as size, shape, amount of fat, and patient movement; image acquisition parameters; specific vendor or machine acquisition, reconstruction, noise reduction, smoothing, and compression techniques; signal-to-noise ratio; and voxel size and shape. For CT, voxel value is measured in Hounsfield units, which are calibrated based on tissue characteristics, with water having a value of 0, and air −1000. For virtually all other medical imaging techniques, no standard gray-scale relationship exists. Raw image data may be acquired in forms uninterpretable by human eyes. Raw k-space data from MRI looks to the eye like a starburst. Commonly, k-space data are transformed via Fourier transform into the images one commonly associates with MRI. There is no reason why ML could not do as well, or better, finding textural patterns in k-space data rather than from typical images that humans view. Raw data manipulation on a similar scale occurs in many other types of medical imaging. Raw datasets often are large. Most raw data are deleted at the time of imaging or soon after based on local disk storage capacity, so for practical purposes datasets of multiple cases of raw data are unavailable.

While texture analysis is widely used for ML image analysis, it is rarely specifically evaluated by radiologists, and seldom annotated or documented in their reports. There are no standards for image texture, and no accepted descriptions of typical anatomic object texture [1].

Most image noise is unstructured, but certain machines or settings generate structured noise. Artifacts occur in every setting; some are dramatic enough that they may be mentioned in radiology reports or otherwise annotated, but in many cases radiologists and technologists simply discount them without mention. Artifacts can be specific to modalities, vendors, individual machines, or even unique situations such as imaging device location (e.g., ICU artifacts on ultrasound) or embedded medical appliances.

## Dataset Specifications—Post Processing

Post processing includes such things as multi-planar reformats or 3D reconstructions; fusion of images from different modalities, time frames, or acquisition parameters; image filtering; segmentation; physiologic function; and time and/or movement analysis of objects. Some but not all post-processing algorithms have been studied for accuracy, reproducibility, and correlation among different vendors [21]. For example, a comparison of four commercially available semiautomatic packages for carotid artery stenosis measurement on CT angiography showed good correlation among the packages, but variable agreement for exact stenosis [22]. Other algorithms such as CT perfusion have shown large value variability among vendors [23].

## Dataset Specifications—Sample Size

Sample size needed to train ML algorithms depends on factors including use case; performance level desired; input features; algorithm type and architecture; number of algorithm parameters; and data quality, including annotation quality, feature distribution, and noise in the training data and extracted features. Larger image matrix size, with more pixels, increases algorithm architecture and requires a larger training sample. The rule of 10 is commonly used, suggesting that a sample size 10 times the number of an algorithm’s parameters is a reasonable estimate of sample size needed for training. Research on estimating data sample size to train ML algorithms is ongoing [24–27].

Depending on the question to be answered and the methodology used, training image analysis ML algorithms may require large datasets. Such a large dataset exists for visible light photographs: ImageNet, the dataset for many computer image recognition competitions, has over 14 million categorized images in 21,000 indexed synsets [28]. Many ImageNet photographs are of low resolution and have relatively low dimensionality.

In contrast, most publically available medical image datasets have tens or hundreds of cases, and datasets with more than 5000 well-annotated cases are rare. In the USA, individual healthcare institutions may have 103 up to rarely 107 of an exam type. These common radiology exam types, for example, chest radiographs, unenhanced brain CTs, mammograms, and abdominal CTs, are often high-dimensional data due to variations in pathology, technique, radiology interpretation, patient population, and clinical setting.

Complete imaging examinations are high-dimensional spaces, and datasets of these examinations provide relatively sparse available data. This is challenging for any method that requires statistical significance, where the needed sample size often grows exponentially with dimensionality. In high-dimensional data, many objects appear to be sparse and dissimilar, which confounds common data organization and search strategies based on detecting areas where objects form groups with similar properties. In addition to absolute sample size, class imbalance affects ML training. This is relevant with medical data if real-world datasets contain only rare target class examples of specific pathology, particularly when that pathology has dramatic clinical consequences and should not be missed.

For tightly focused use cases and algorithms with relatively few parameters, however, sample sizes as small as hundreds of cases may be adequate for training [4]. Because of this, much current academic medical image analysis ML research occurs this way.

The required number of unique, original exams can be decreased with hybrid approaches using augmentation and/or pre-training with selected features. Augmentation uses techniques to modify the dataset while keeping the key features the algorithm needs. For images, this commonly involves rotating, mirroring, or adjusting contrast or grayscale of images or parts of images such as a mass or other pathology, to generate new images from originals. For radiology examinations, algorithms may be pre-trained on other image datasets, even non-medical ones, in a process known as transfer learning [5, 29].

## Medical Image Dataset Specifications—Regulatory and Business

In the USA, HIPAA mandates that unless specifically authorized by the patient to release patient-identifiable data, all PHI must be removed. HIPAA specifies 18 different identifiers that must be removed, including “full-face photographs and any comparable images.” Depending on the body part imaged, 3D reconstruction of radiology exams into parts of a patient’s image is possible. On the other hand, de-identification of datasets may result in the removal of metadata that is required for advanced processing.

The NIH requires applicants/grantees for most studies to submit a plan to share their final research data, and encourages all applicants to include a plan to address data sharing or to state why data sharing is not possible [10, 30]. Datasets may be shared by permitting access to a specific source or by sharing datasets through NIH data repositories [31]. Despite these requirements, applicable medical image datasets may not be readily available. C-MIMI participants universally felt that NIH should enforce adherence to data sharing regulations.

Though radiology societies express interest to develop medical image dataset production, storage, and management infrastructure, there is not yet a viable business case for them given the expense and resource requirements to build and maintain.

The FDA’s Medical Device Development Tools (MDDT) program is a way for the FDA to qualify tools that medical device sponsors can use in the development and evaluation of medical devices. Qualification means that the FDA has evaluated the tool and concurs with available supporting evidence that the tool produces scientifically plausible measurements and works as intended within the specified context of use [32]. The FDA may be receptive to data submission to FDA for incorporation into the biomarker MDDT pilot, and/or submit tools to manage datasets appropriate for ML.

For individual healthcare enterprises, patient privacy, business case, and logistical issues are barriers to releasing or otherwise using medical images. As these institutions realize the value of those data, and the potential value of participating in ML model development, some of them are starting to pursue business relationships to expose data to data science businesses outside their firewalls. These same issues add complexity to any proposed large, federated, commercially or publically available medical image datasets with exams acquired from disparate healthcare institutions. Other countries with more centralized image storage, interoperable medical image management, and different regulatory and legal environments may provide lower barriers to data access.

Because of HIPAA and legal requirements on data privacy, as well as intellectual property issues, academic and other healthcare settings are starting to host, test, and do research with commercial algorithms inside their firewalls. This concept of bringing algorithms to the data, rather than data to the algorithm, is rapidly gaining favor. It allows these settings to keep control over their data, and still permit industry to move forward. One consideration with this approach is if institutions should keep back some of the data to use for testing, validation, and verification and, if so, how it is managed.

Who owns radiology data is not legally defined in most states. HIPAA does not specify, and most states have no legislation regarding medical data ownership [33]. Obtaining patient consent for secondary reuse of clinical data is often difficult and impractical.

## Medical Image Dataset Specifications—Cataloging and Discovery

Widely used standards to catalog and discover medical image datasets do not exist, nor does a framework to catalog datasets specific to ML. Currently, mechanics of dataset discovery, collection, curation, access, and use are typically one-off solutions related to a specific research project. Medical multimedia metadata are often dirty and inconsistent. Some require high-level-domain expertise to understand, and others may require institutional knowledge unique to the originating site. Table 2 describes high-level metadata needed to catalog and discover medical image datasets. Many of these metadata are semantic. For example, under image type, if the modality is MRI, further data ideally would include details such as machine vendor, machine strength, model number, coil details, and contrast type and amount.

**Table 2:** Baseline metadata to catalog medical image data

1. Image types
a. Modality
b. Resolution
c. Number of images total and by series
2. Number of imaging examinations
3. Image examination source(s)
4. Image acquisition parameters
5. Image storage parameters (e.g., compression amount and type)
6. Annotation
a. Type
b. What is annotated, and how
7. Context
8. How is ground truth defined and labeled
9. Associated data
a. Demographic
b. Clinical
c. Lab
d. Genomic
e. Timeline
f. Social media
10. Date range of image exam acquisition
11. Log of dataset use
12. Who owns the data
13. Who is responsible for the data
14. Allowable usage
15. Access parameters
a. Accessibility
b. Costs and business agreements
16. Case distribution
a. % Normals vs abnormals
b. Summary of abnormal examinations
i. Number of examinations with each pathology

Many of these are semantic, with further subcategories not listed here

Recently, the scientific data community has proposed the FAIR guiding principles to support reuse of scholarly data [2, 34]. These guiding principles—Findability, Accessibility, Interoperability, and Reusability—should be no different for medical imaging ML datasets and are summarized in Table 3. Additionally, there is good reason to suggesting that the FAIR principles should apply not only to ML datasets but also to tools, algorithms, and workflows created for ML.

**Table 3:** The FAIR guiding principles for scientific data management—Findability, Accessibility, Interoperability, and Reusability (https://www.ncbi.nlm.nih.gov/pmc/articles/PMC4792175/, Creative Commons License)

The FAIR guiding principles
To be Findable:
F1. (Meta)data are assigned a globally unique and persistent identifier.
F2. Data are described with rich metadata (defined by R1 below).
F3. Metadata clearly and explicitly include the identifier of the data it describes.
F4. (Meta)data are registered or indexed in a searchable resource.
To be Accessible:
A1. (Meta)data are retrievable by their identifier using a standardized communications protocol.
A1.1 The protocol is open, free, and universally implementable.
A1.2 The protocol allows for an authentication and authorization procedure, where necessary.
A2. Metadata are accessible, even when the data are no longer available.
To be Interoperable:
I1. (Meta)data use a formal, accessible, shared, and broadly applicable language for knowledge representation.
I2. (Meta)data use vocabularies that follow FAIR principles.
I3. (Meta)data include qualified references to other (meta)data.
To be Reusable:
R1. (Meta)data are richly described with a plurality of accurate and relevant attributes.
R1.1. (Meta)data are released with a clear and accessible data usage license.
R1.2. (Meta)data are associated with detailed provenance.
R1.3. (Meta)data meet domain-relevant community standards.

## Summary

The dominant influence on a machine learning (ML) model’s performance is often the amount and quality of training data. Attendees of the inaugural C-MIMI expressed the common theme that everyone participating in medical image evaluation with ML is data starved. There is an urgent need to find ways to collect, annotate, discover, and, ideally, reuse adequate amounts of medical imaging data.

Action items, and priority research topics, for this field of study include the following:

- Describe, via a whitepaper, the high-level attributes of reusable medical image datasets suitable to train, test, validate, verify, and regulate ML products [35]
- Describe common categories of use cases for medical image datasets, and understand unique dataset attributes applicable to each
- Describe the metadata, framework, and standards needed to catalog and discover datasets of medical images appropriate for ML.
- Understand and describe business cases and models for medical image datasets
- Work with NIH and other government agencies to promote and, where applicable, enforce, access to medical image datasets
- Improve communication among medical imaging domain experts, medical imaging informaticists, government and industry data scientists, and interested commercial entities

At the second annual Conference on Machine Intelligence in Medical Imaging (C-MIMI) meeting, to be held on September 26–27, 2017, at Johns Hopkins in Baltimore, Maryland, and at the SIIM Annual Meeting on June 1–3, 2017, in Pittsburgh, Pennsylvania, these action items will be reviewed further, as well as continuing the discussion on medical image datasets for ML.
